# Three-dimensional periodontal tissue regeneration using a bone-ligament complex cell sheet

**DOI:** 10.1038/s41598-020-58222-0

**Published:** 2020-02-03

**Authors:** Resmi Raju, Masamitsu Oshima, Miho Inoue, Tsuyoshi Morita, Yan Huijiao, Arief Waskitho, Otto Baba, Masahisa Inoue, Yoshizo Matsuka

**Affiliations:** 1 Department of Stomatognathic Function and Occlusal Reconstruction, Graduate School of Biomedical Sciences, Tokushima University Tokushima, 770-8503 Japan; 2 Department of Oral and Maxillofacial Anatomy, Graduate School of Biomedical Sciences, Tokushima University Tokushima, 770-8503 Japan; 3 Laboratories for Structure and Function Research, Faculty of Pharmaceutical Sciences, Tokushima Bunri University, Tokushima, 770-8055 Japan

**Keywords:** Regenerative medicine, Tissue engineering

## Abstract

Periodontal tissue is a distinctive tissue structure composed three-dimensionally of cementum, periodontal ligament (PDL) and alveolar bone. Severe periodontal diseases cause fundamental problems for oral function and general health, and conventional dental treatments are insufficient for healing to healthy periodontal tissue. Cell sheet technology has been used in many tissue regenerations, including periodontal tissue, to transplant appropriate stem/progenitor cells for tissue regeneration of a target site as a uniform tissue. However, it is still difficult to construct a three-dimensional structure of complex tissue composed of multiple types of cells, and the transplantation of a single cell sheet cannot sufficiently regenerate a large-scale tissue injury. Here, we fabricated a three-dimensional complex cell sheet composed of a bone-ligament structure by layering PDL cells and osteoblast-like cells on a temperature responsive culture dish. Following ectopic and orthotopic transplantation, only the complex cell sheet group was demonstrated to anatomically regenerate the bone-ligament structure along with the functional connection of PDL-like fibers to the tooth root and alveolar bone. This study represents successful three-dimensional tissue regeneration of a large-scale tissue injury using a bioengineered tissue designed to simulate the anatomical structure.

## Introduction

Periodontal tissue is a specialized three-dimensional multicellular structure composed of cementum, periodontal ligament (PDL) and alveolar bone^1^. PDL can cooperate with the maxillofacial region through fibrous connections with the cementum and the alveolar bone, and it plays important roles in biological functions, including the absorption of occlusal forces, maintenance of alveolar bone height and tooth movement via bone remodelling^2,3^. Periodontal diseases cause fundamental problems for oral function due to a severe inflammatory reaction caused by bacterial infection or traumatic force^4^. Progressive periodontal disease can cause irreversible changes in the affected area, and if not treated it leads to tooth loss, dysfunction and thereby affects the quality of life^5^. Conventional dental treatments for periodontal disease, such as scaling and root planing, have been widely utilized in clinical practice to mechanically remove the source of the infection. However, it commonly results in the formation of long junctional epithelium on the root surface and the damaged periodontal tissue is difficult to heal with only these mechanical approaches^6^. To regenerate healthy periodontal tissue, various clinical techniques were developed to prevent downward epithelial migration and promote periodontal tissue regeneration by the remaining PDL cells or osteoblasts. Guided tissue regeneration (GTR) and guided bone regeneration (GBR) have been used in various membranes to create a three-dimensional space between the defect and root/bone surface to allow the repopulation of cells, and thereby regeneration of PDL and bone tissue. Although these techniques are effective in current dental treatment, a long-term stable clinical outcome has not been attained^7,8^.

Recent advances in regenerative medicine have been supported by studies in embryonic development, stem-cell biology and tissue engineering technology^9,10^. Stem-cell transplantation and cytokine therapy, which target structural and functional defects are considered regenerative concepts for the repair of damaged tissues^11,12^. In the dental research field, tooth-tissue derived stem cells, adipose-derived stem cells and cytokines have been well characterised, and the information can be applied to the repair of periodontal tissue components such as cementum, PDL and alveolar bone^13,14^. PDL-derived stem cells (PDLSCs) have been identified in adult human PDL tissue, and are able to differentiate into all of the periodontal tissue components and generate cementum and the PDL complex structure after *in vivo* transplantation^2,15^. Dental follicle stem cells (DFSCs) were identified as mesenchymal stem/progenitor cells in the first molars of neonatal rat, and they can differentiate into osteoblasts, cementoblasts, adipocytes, and neural cells^16–18^. Moreover, gingival tissue has been identified as a source of mesenchymal stem cells with the characteristics such as homogeneity of cells, easy to isolate and faster proliferation rate, which makes them a promising source for the regeneration field^19–21^. All these stem cells are considered a candidate cell source for periodontal tissue regeneration, and several molecular treatments can be administered via local application of recombinant cytokines, such as fibroblast growth factor-2 (FGF-2), transforming growth factor-β1 (TGF-β1) and bone morphogenetic protein (BMP)^22–24^, to repair periodontal tissue injury. In addition, enamel matrix derivative (EMD) has been applied clinically to restore periodontal tissues^25,26^. These molecular treatments have great potential for periodontal tissue regeneration through the stimulation of signaling pathways associated with cell proliferation and differentiation. However, the limitation of cell transplantation and cytokine therapy includes the difficulty in delivering and stabilizing a sufficient quantity of cells/molecules into the defect area^13^. Furthermore, tissue engineering is an attractive approach introduced in the field of periodontal tissue regeneration. Tissue engineering utilizes various porous scaffolds made of biomaterials to regenerate periodontal tissue around tooth and dental implants^27–29^.

Cell sheet engineering with a temperature responsive culture dish has various advantages over the regenerative methods using artificial scaffolds. Cells can be harvested as a single sheet without destroying the cellular attachment proteins and extracellular matrix^30^. Scaffold-free cell sheet engineering has been applied in regeneration of such tissues as cornea, heart, esophagus, cartilage, liver and periodontal^31^. A PDL cell sheet for the regeneration of periodontal tissue is now at the stage of clinical therapy for periodontal disease^32^. Although this technology contributes to partial tissue repair, a single cell sheet cannot sufficiently regenerate a large-scale tissue injury^33^. To solve this problem, some researchers have tried to use a combination of multiple cell sheets and artificial scaffolds including more than one type of cell^34–38^. Even though these biomaterials have shown preliminary successful regeneration of periodontal tissue, they still include a non-living artificial material, and the long-term biological response following the implantation of such materials are still questionable^37^. An ideal goal of tissue engineering technologies is the application of a well-designed bioengineered tissue without artificial materials that simulate anatomical structure, to enable a complete three-dimensional tissue regeneration for a large-scale tissue injury area^38^.

In this study, we demonstrated a three-dimensional regeneration of periodontal tissue using a complex cell sheet composed of PDL cells and osteoblast-like cells. Our complex cell sheet anatomically reproduced bone-ligament structure equivalent to natural periodontal tissue in *in vivo* transplantation. This study highlights a successful three-dimensional tissue regeneration of a large-scale tissue injury using a bioengineered tissue designed to simulate the anatomical structure.

## Results

### *In vitro* characteristics of cells

**Figure 1:**
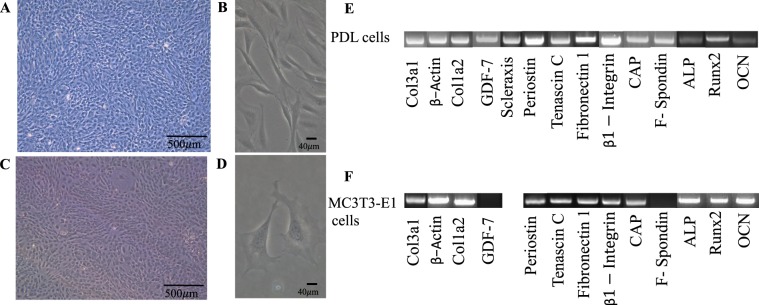
Cell culture characteristics and identification of cells. (**A**,**B**) PDL cell culture at low and high magnification at passage 3, (**C**,**D**) MC3T3-E1 cell culture at low and high magnification, (**E**) Gene expression patterns in rat PDL cells, (**E**) Gene expression patterns in MC3T3-E1 cells.

We have used two types of cells for the fabrication of cell sheets, which includes, rat PDL cells and osteoblast like cells (MC3T3-E1cells). PDL cells were isolated from the extracted molars of 4–5 weeks old SD rats. Mouse calvaria derived MC3T3-E1 cells were obtained from Riken industry, Japan. The cell morphology was identified under a light microscope. Rat PDL cells exhibited a fusiform and spindle shaped morphology (Fig. 1A,B). MC3T3-E1 cells showed a polygonal and fusiform shape (Fig. 1C,D). Semi-quantitative PCR and agarose gel electrophoresis was carried out to identify the genes related to each cell type. Rat PDL cells exhibited a positive expression related to the collagen type 3, alpha 1 (col3a1), β-actin, collagen type I, alpha 2 (col1a2), growth differentiation factor 7 (GDF-7), scleraxis, periostin, tenascin C, fibronectin 1, β1-integrin, cementum attachment protein (CAP) and f-spondin, low intensity bands were observed for alkaline phosphatase (ALP), runt related transcription factor-2 (Runx-2) and osteocalcin (OCN) genes by RT-PCR and agarose gel electrophoresis (Fig. 1E). Similarly, MC3T3-E1 cells showed positive gene expression for col3a1, β-actin, col1a2, periostin, tenascin C, fibronectin 1, β1-integrin, CAP, ALP, Runx-2 and OCN. Furthermore, GDF-7 and f-spondin did not show any expression for MC3T3-E1 cells(Fig. 1F).

### *In vitro* analysis of three different cell sheets

**Figure 2:**
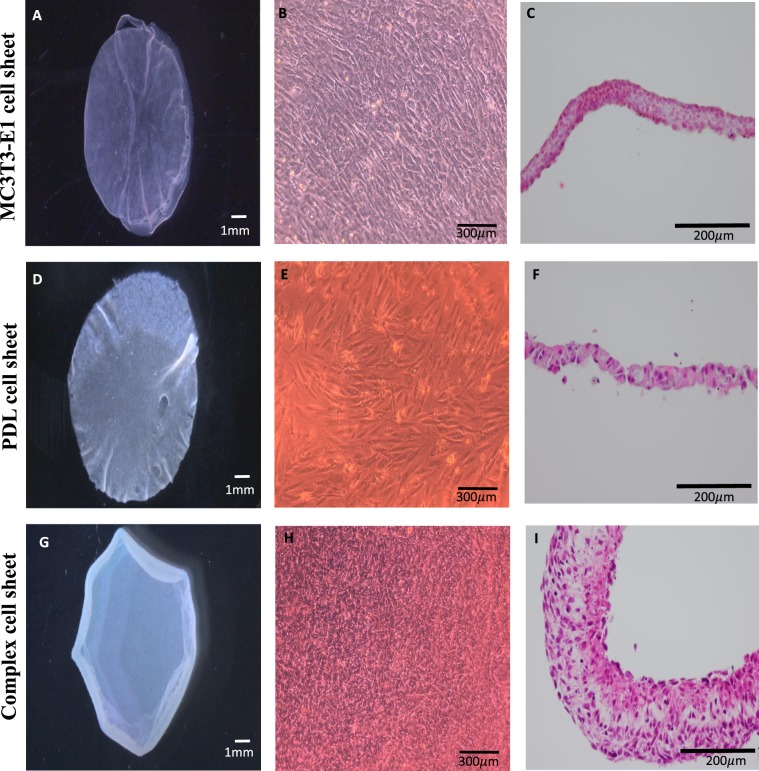
Fabrication of cell sheets by temperature responsive culture dishes. (**A**) MC3T3-E1 cell sheet, (**B**) MC3T3-E1 cell culture in temperature responsive culture well, (**C**) H&E staining of MC3T3-E1 cell sheet, (**D**) PDL cell sheet, (**E**) PDL cell culture in temperature responsive culture well, (**F**) H&E staining of PDL cell sheet, (**G**) Complex cell sheet, (**H**) Complex cell sheet culture in temperature responsive culture well, (**I**) H&E staining of complex cell sheet.

**Figure 3:**
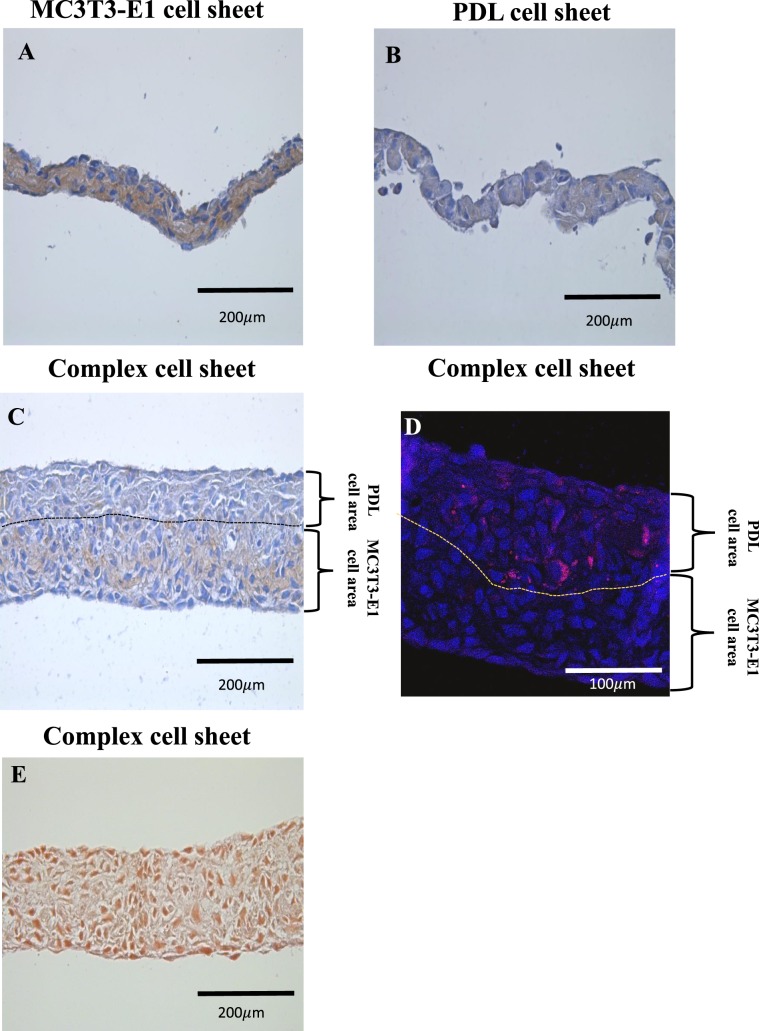
Immunohistochemical staining, species specific FISH probe staining and alizarin red staining of complex cell sheet. (**A**) MC3T3-E1 cell sheet showed positive staining for anti-mouse-OCN antibody, (**B**) PDL cell sheet did not show any positive staining for anti-mouse-OCN antibody, (**C**) Complex cell sheet showed positive expression for anti-mouse-OCN antibody in MC3T3-E1 cells space and negative staining in the PDL cells area, PDL cells and MC3T3-E1 cells area is demarcated using black dotted lines, (**D**) Rat species specific FISH probe stained complex cell sheet shows positively expressed rat PDL cells in red color, cells counterstained with DAPI are shown in blue, PDL cells and MC3T3-E1 cells area is demarcated using yellow dotted lines, (**E**) Alizarin red staining of complex cell sheet did not show any mineral deposition.

Three types of cell sheets were prepared for the study (Supplementary data, Fig. S1), two single cell sheets containing either PDL cells (PDL cell sheet) or MC3T3-E1 cells (MC3T3-E1 cell sheet) and a complex cell sheet containing both cells. All cell sheets were fabricated by culturing in a temperature responsive culture dish. The morphology of the cell sheets was observed using light microscope (Fig. 2B,E,H), and a stereomicroscope (Fig. 2A‚D,G). The complex cell sheet (Fig. 2G) appeared thicker than either the MC3T3-E1 cell sheet (Fig. 2A) or PDL cell sheet (Fig. 2D). The marginal edges of the complex cell sheets were rolled in toward the side of the PDL cells layer (Fig. 2G). This was an advantageous identification in our study, which was later used to orient the PDL cell side towards the tooth root in ectopic and orthotopic transplantation of cell sheets. H&E staining of the cell sheets revealed that the MC3T3-E1 cell sheet and PDL cell sheets were composed of 3 to 4 layers of cells (Fig. 2C,F). Alternatively, the complex cell sheets resulted in around 10 layers of cells (Fig. 2I). Furthermore, to identify distinct layers of 2 types of cells in complex cell sheet, we performed immunohistochemistry using anti-mouse-OCN antibody and species specific fluorescent *in situ* hybridization (FISH) probe for rat cells. Immunohistochemical analysis of cell sheets by anti-mouse-OCN antibody shows a positive expression in MC3T3-E1 cell sheet (Fig. 3A) and the MC3T3-E1 cell area in complex cell sheet (Fig. 3C, PDL cells and MC3T3-E1 cells area is demarcated using black dotted lines). PDL cell sheet and PDL cell area in complex cell sheet did not express a positive staining against anti-mouse-OCN antibody (Fig. 3B,C). As we have used anti-mouse-OCN antibody, the negative expression in rat PDL cell layer does not necessarily indicate the absence of OCN expression by PDL cells. Furthermore, species specific FISH probe for rat species showed an immunoreactivity against rat PDL cells and there was a negative reaction in MC3T3-E1 cell side in complex cell sheet (Fig. 3D, PDL cells and MC3T3-E1 cells area is demarcated using yellow dotted lines). Thereby, the immunohistochemical staining against anti-mouse-OCN antibody and species specific FISH probe for rat species showed the distinct layers of mouse and rat cell area in complex cell sheet (Fig. 3C,D). In addition, we have done alizarin red staining of complex cell sheets to evaluate the formation of any mineralized nodules. Complex cell sheets did not form any mineral nodules prior to the *in vivo* transplantation of cell sheet (Fig. 3E).

### The complex cell sheet promoted the generation of periodontal tissue in ectopic transplants

**Figure 4:**
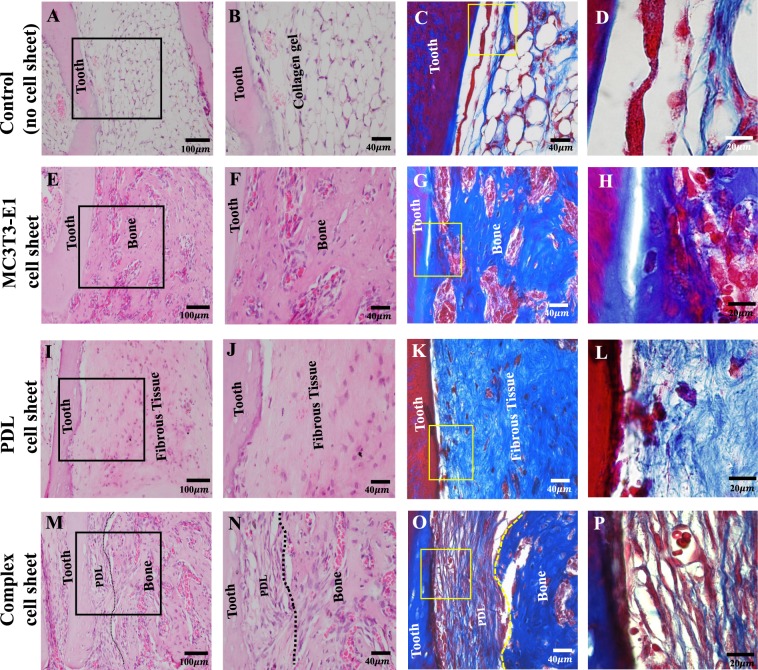
H&E and azan staining of ectopic transplants. (**A–D**) Control, without any cell sheet transplantation shows only collagen gel structure around the tooth, (**E–H**) MC3T3-E1 cell sheet transplants showed only bone like tissue formation, (**I–L**) PDL cell sheet transplants with only fibrous tissue generation, (**M–P**) Complex cell sheet transplants showed both PDL and bone-like tissue (PDL-like and bone-like tissue is demarcated using black and yellow dotted lines).

We next investigated whether the cell sheets could generate periodontal tissue around the carrier tooth root after ectopic transplantation in the subrenal capsule of immunocompromised mice. We used extracted, enzymatically digested and autoclaved mouse mandibular tooth (devoid of any cells) as a carrier to support the cell sheet. The carrier tooth was enveloped with the cell sheet, inserted into a collagen gel and the construct including carrier tooth, cell sheet and collagen gel was then transplanted into the subrenal capsule of mice (Supplementary data, Fig. S2). Four groups were included for ectopic transplantation. The control group (n = 4) was a tooth without a cell sheet. The treatment groups were a MC3T3-E1 cell sheet (n = 4), PDL cell sheet (n = 10) or complex cell sheet (n = 10) wrapped around the carrier tooth root and embedded in collagen gel. After 4 weeks of ectopic transplantation of cell sheets, we harvested the specimens and analyzed the generated tissue morphological characteristics using H&E staining, azan staining and immunohistochemistry under light microscope. H&E and azan staining revealed the presence of collagen gel structure around the carrier tooth without any tissue formation in the control group that was transplanted without any cell sheets (Fig. 4A–D). Bone-like tissue attached directly onto the tooth root was observed in the MC3T3-E1 cell sheet group (Fig. 4E–H), whereas the PDL cell sheet group produced an irregularly arranged fibrous tissue and weak attachment of this tissue to the tooth root (Fig. 4I–L). Alternatively, a combination of PDL-like and bone-like tissue formation was evident in the complex cell sheet group (Fig. 4M,N). There was a clear demarcation between PDL and bone-like tissue in the complex cell sheet group (shown by black dotted lines in Fig. 4M,N). Using azan staining, we have observed PDL-like fibers and newly formed bone-like tissue in the complex cell sheet group (Fig. 4O,P, PDL-like and bone-like tissue demarcation is shown by yellow dotted lines in Fig. 4O).

**Figure 5:**
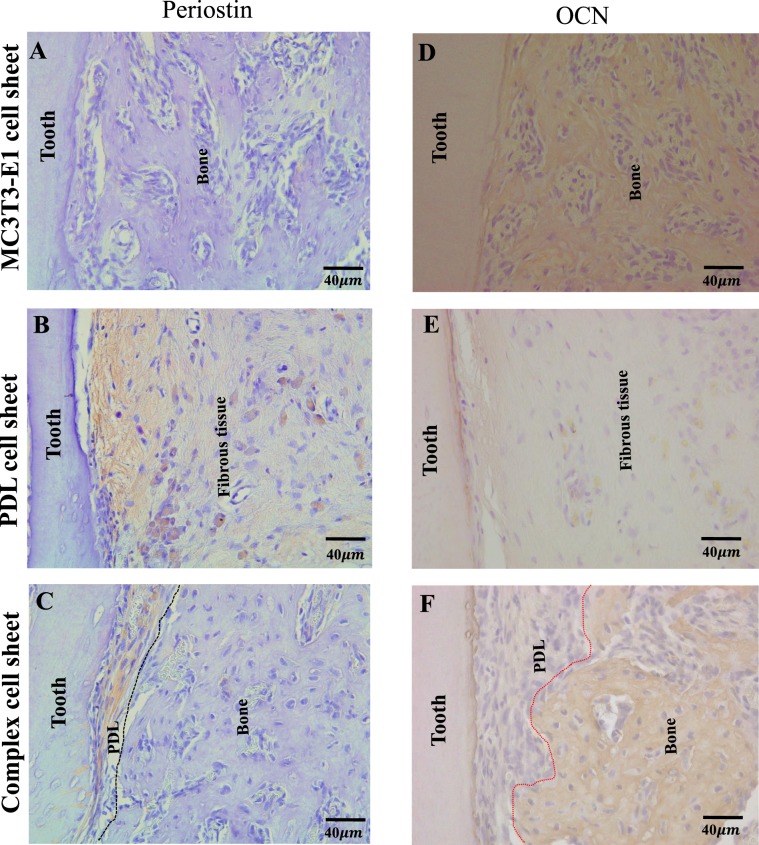
Immunohistochemical staining of ectopic transplants. (**A**) MC3T3-E1 cell sheet transplants showed negative staining for anti-periostin antibody, (**B**) PDL cell sheet transplants shows positive staining for anti-periostin antibody, (**C**) Complex cell sheet transplants showed positive staining for anti-periostin antibody in the PDL-like tissue space (PDL-like and bone-like tissue is demarcated using black dotted lines), (**D**) MC3T3-E1 cell sheet transplants showed positive staining for anti-mouse-OCN antibody, (**E**) PDL cell sheet transplants did not show positive staining for anti-mouse-OCN antibody, (**F**) Complex cell sheet transplants showed positive OCN staining at the bone-like tissue area (PDL-like and bone-like tissue is demarcated using red dotted lines).

Furthermore, we performed immunohistochemistry analysis using anti-periostin and anti-mouse-OCN antibody to confirm the formation of PDL-like and bone like tissue. Immunohistochemistry staining revealed the expression of periostin in PDL cell sheet group (Fig. 5B) as well as in the PDL-like tissue area of complex cell sheet group (Fig. 5C). Moreover, periostin expression was lacking in transplants from MC3T3-E1 group (Fig. 5A) and the bone-like tissue area in complex cell sheet transplants (Fig. 5C, tissue demarcation is indicated using black dotted lines). On the other hand, immunohistochemistry staining using anti-mouse-OCN antibody revealed the localization of OCN in the MC3T3-E1 and complex cell sheet groups (Fig. 5D‚F). The MC3T3-E1 cell sheet transplants showed ankylosis of the tooth root with direct attachment of bone tissue and an absence of fibers attached to the tooth root (Fig. 5D). Complex cell sheet group revealed positive OCN expression in the bone-like tissue generated area (Fig. 5F, PDL-like and bone-like tissue demarcation is indicated using red dotted lines). As the PDL cell sheet group and PDL-like tissue area in complex cell sheet group is generated from rat PDL cells, it lacked positive reaction towards the anti-mouse-OCN antibody (Fig. 5E,F).

Complex cell sheet transplants revealed the expression of periostin in PDL-like tissue generated area and OCN expression in bone-like tissue generated area, which in turn indicates the formation of periodontal tissue like structure following ectopic transplantation of complex cell sheet.

### Complex cell sheet promotes the regeneration of periodontal tissue in orthotopic transplants

Finally, to analyze the regenerative capacity of cell sheets in an orthotopic model, we created a periodontal tissue injury model mouse to accommodate the cell sheet. Injury was confining to the palatal surface of maxillary first molar tooth. We removed the periodontal ligament fibers and bone and created the defect with a size of 2 mm in length (anterior-posterior direction), 0.5 mm in width (buccal-palatal direction) and 1.5 mm in depth (Supplementary data, Fig. S3A,B). Five groups were categorized in orthotopic transplantation to observe periodontal tissue regeneration. The groups were natural periodontal tissue (n = 4), a periodontal tissue injury model (n = 4), control group (without any cell sheet transplantation) after 8 weeks (n = 6), a PDL cell sheet transplanted group after 8 weeks (n = 6) and complex cell sheet transplanted group after 8 weeks (n = 6).

**Figure 6:**
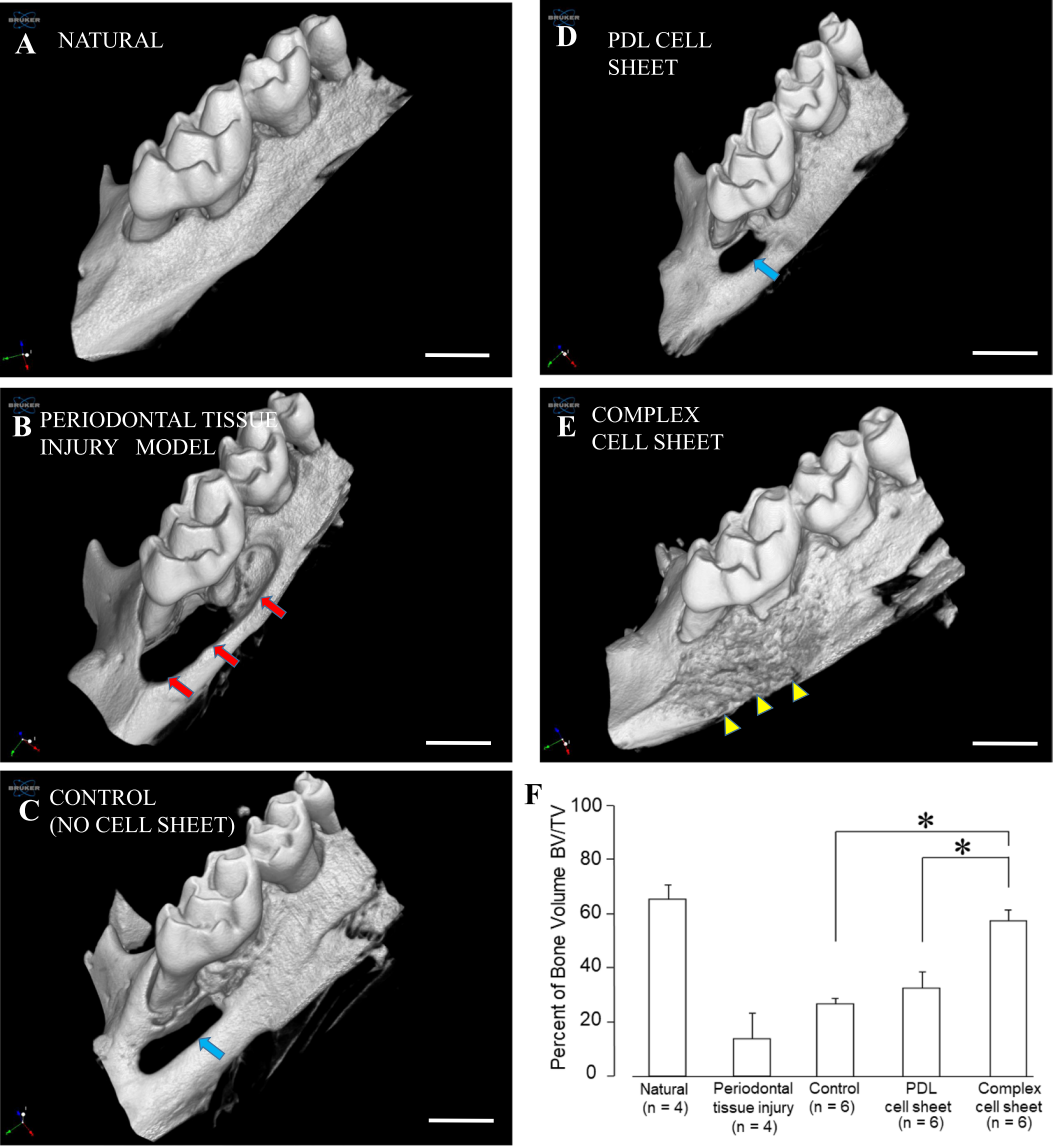
Three-dimensional micro-CT images and regenerated bone volume analysis of orthotopic periodontal tissue injury models. (**A**) Natural periodontal tissue area, (**B**) Periodontal tissue injury created on the palatal surface of maxillary first molar (Red arrow shows the injury area), (**C**) Control, without any cell sheets transplantation after 8 weeks (blue arrow shows the area which is not regenerated), (**D**) PDL cell sheet transplant following 8 weeks (blue arrow shows the area which is not regenerated), (**E**) Complex cell sheet transplant following 8 weeks (yellow arrowhead shows the regenerated area), (**F**) Percent of Bone volume/ Tissue volume (BV/TV) in the natural periodontal tissue, periodontal tissue injury area, control group after 8 weeks (without any cell sheets), PDL cell sheet transplanted area after 8 weeks and complex cell sheet transplanted site after 8 weeks. The data indicates that there is a significant difference in the bone volume in control and PDL cell sheet group compared to complex cell sheet group. In contrast, significant difference was not observed in natural periodontal tissue group and complex cell sheet group. Error bar represent the standard deviation. *p < 0.05 (One-way ANOVA, Tukey HSD test). Scale bar indicates 1 mm.

The three-dimensional images of micro-CT evaluation demonstrated the natural periodontal tissue structure near the palatal surface of maxillary first molar tooth (Fig. 6A), periodontal tissue injury model on the palatal surface of maxillary first molar tooth (Fig. 6B, red arrow shows the periodontal tissue injury area), control (without any cell sheet transplantation) after 8 weeks (Fig. 6C) and the PDL and complex cell sheet transplant groups after 8 weeks (Fig. 6D,E). There was incomplete regeneration of mineralized tissue in the control group transplants (Figs. 6C and S4, blue arrow shows the area which is not regenerated) and PDL cell sheet group (Figs. 6D and S4, blue arrow shows the area which is not regenerated) after 8 weeks of orthotopic transplantation. Alternatively, a complete recovery of periodontal tissue injury with a newly formed mineralized tissue was observed in the complex cell sheet transplanted group (Figs. 6E and S4, yellow arrow heads), which was similar to the natural periodontal tissue (Fig. 6A). Furthermore, the regenerated bone volume (bone volume/tissue volume; BV/TV) at the periodontal tissue injury was calculated using the reconstructed three dimensional images. There was no significant difference in bone volume between complex cell sheet group and natural periodontal tissue group. In contrast, there was a significant difference in the bone volume in PDL cell sheet and control group compared to the complex cell sheet group (Fig. 6F). Abundant formation of mineralized tissue with an appropriate PDL space was observed in three-dimensional coronal sections at the anterior, middle and posterior portions of the tooth and the injury site in the complex cell sheet transplanted group (Fig. S5E, yellow arrow heads) similar to the normal structure (Fig. S5A). However, partial recovery of the defects with mineralized matrix was observed in the transplants from control group (Fig. S5C) and PDL cell sheet groups following 8 weeks (Fig. S5D).

**Figure 7:**
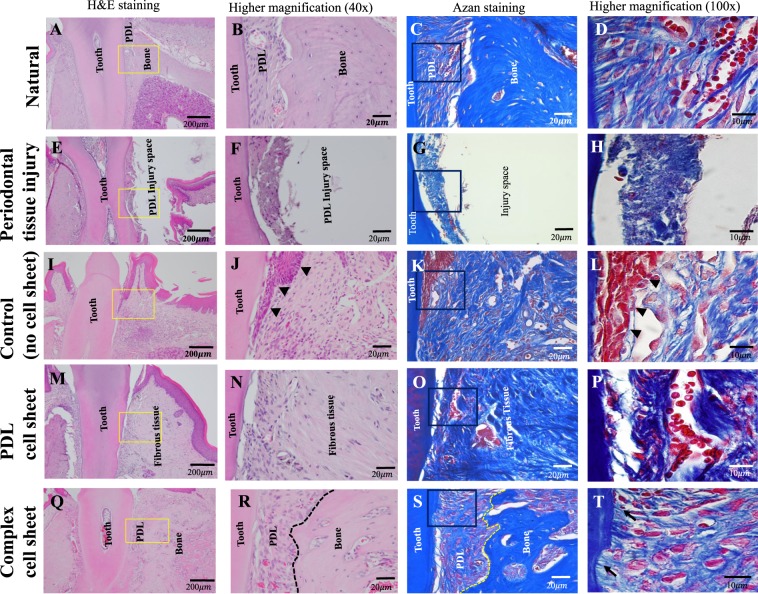
H&E and azan staining of orthotopic periodontal tissue injury site. (**A–D**) Natural periodontal tissue without any periodontal tissue injury, (**E–H**) Periodontal tissue injury, (**I–L**) Control, without any cell sheet transplantation after 8 weeks (black arrow heads show the epithelial tissue migration to the root surface) (**M–P**) PDL cell sheet transplant after 8 weeks, (**Q–T**) Complex cell sheet transplant after 8 weeks (PDL-like and bone-like tissue is demarcated using black dotted lines in H&E and yellow dotted lines in azan staining, black arrow points show the PDL fibers attached similar as in natural periodontal tissue to the root respectively).

Histological images of the natural architecture of periodontal tissue and an injury model created on day 1 by removing the PDL and bone tissue are shown in Fig. 7A–H.

An evident downward growth of epithelial tissue layer with the absence of any bone like tissue formation was observed with the H&E and azan staining of the transplants from the control group (Fig. 7I–L, shown in black arrow heads). There was an irregular arrangement of fibrous tissue with the absence of bone-like tissue formation was observed in the PDL cell sheet group (Fig. 7M–P). H&E staining revealed PDL-like fibers and bone-like tissue formation in the complex cell sheet group (Fig. 7Q,R). Azan staining revealed the functional connection of PDL-like fibers to the root surface equivalent to the natural PDL (Fig. 7S,T, shown in black arrows). Furthermore, there was a clear demarcation between the PDL space and bone-like tissue formation similar to natural periodontal tissue (Fig. 7R,S, separated with a black dotted line in the H&E image and yellow dotted line in the azan staining image).

**Figure 8:**
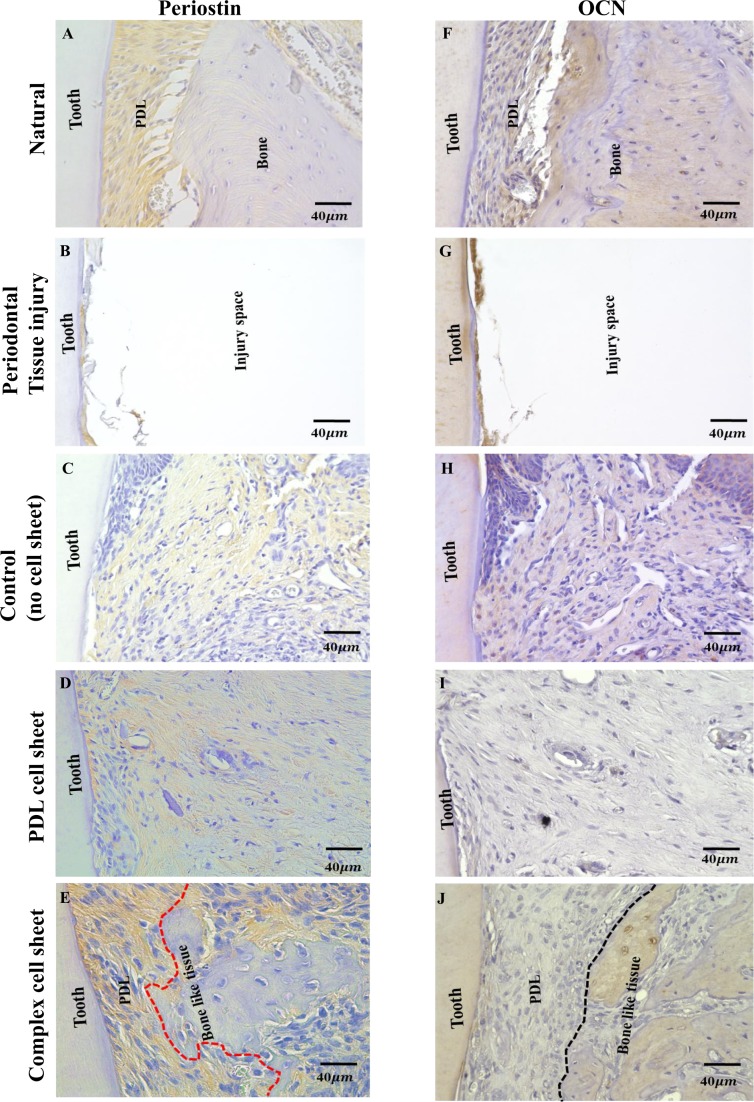
Immunohistochemical staining of orthotopic transplants. (**A**) Natural periodontal tissue shows positive staining for anti-periostin antibody in the PDL area, (**B**) Periodontal tissue injury shows a negative staining for anti-periostin antibody, (**C**) Control, without any cell sheet transplantation after 8 weeks shows a weak positive staining for anti-periostin antibody, (**D**) PDL cell sheet transplant after 8 weeks shows a weak positive staining for anti-periostin antibody, (**E**) Complex cell sheet transplant after 8 weeks shows positive staining for anti-periostin antibody in the PDL space and negative staining in newly formed bone-like tissue (PDL-like and bone-like tissue is demarcated using red dotted lines) (**F**) Natural periodontal tissue shows positive staining against anti-mouse-OCN antibody in PDL as well as bone tissue, (**G**) Periodontal tissue injury shows no significant staining, (**H**) Control group, without any cell sheets after 8 weeks shows negative staining for anti-mouse-OCN antibody, (**I**) PDL cell sheet transplant after 8 weeks negative staining for anti-mouse-OCN antibody, (**J**) Complex cell sheet transplant after 8 weeks shows positive staining for anti-mouse-OCN antibody in the bone-like tissue area (PDL-like and bone-like tissue is demarcated using black dotted lines).

Finally, we performed immunohistochemistry analysis using anti-periostin and anti-mouse-OCN antibody to confirm the formation of PDL-like and bone-like tissue formation in the orthotopic transplants. Immunohistochemistry staining revealed the presence of periostin in PDL of natural periodontal tissue (Fig. 8A). There was no significant staining against anti-periostin antibody in periodontal tissue injury model (Fig. 8B). Moreover, a weak periostin expression was observed in transplants from control group and PDL cell sheet group (Fig. 8C,D). Furthermore, a strong expression against anti-periostin antibody in the PDL area and negative expression of periostin in the regenerated bone tissue area was observed in complex cell sheet group (Fig. 8E, tissue demarcation is indicated using red dotted lines). In contrast, immunohistochemistry staining using anti-mouse-OCN antibody revealed the presence of OCN in the PDL as well as bone tissue in natural periodontal tissue (Fig. 8F). No significant staining was observed in periodontal tissue injury model (Fig. 8G). The transplants from control group lacked any positive reaction towards anti-mouse OCN antibody (Fig. 8H). As the PDL cell sheet group and PDL-like tissue area in complex cell sheet group is regenerated from rat PDL cells, it lacked positive reaction towards the anti-mouse-OCN antibody (Fig. 8I,J). Alternatively, the complex cell sheet group revealed positive OCN expression in the bone-like tissue regenerated area (Fig. 8J, PDL-like and bone-like tissue demarcation is indicated using black dotted lines). These results indicate the regeneration of three-dimensional periodontal tissue like structure equivalent to the architecture of layered bone-ligament construct/ complex cell sheet.

## Discussion

In this study, we fabricated a complex cell sheet composed of PDL cells and osteoblast-like cells that reproduce the bone-ligament structure equivalent to natural periodontal tissue. We also demonstrated a three-dimensional regeneration of periodontal tissue using a complex cell sheet in ectopic and orthotopic transplantation. This study represents the feasibility of a three-dimensional tissue engineering/regeneration from an anatomically designed bioengineered complex tissue.

Cell sheet technology has a great advantage in that it can transplant appropriate stem/progenitor cells for tissue regeneration of target site as a uniform tissue, and can be used clinically for diseases and injuries in various tissues^31,32^. Different types of cell sheets, such as cartilage, esophagus epithelium and myocardium, can be easily made using tissue-derived stem/progenitor cells^39–41^, and a current advance in this field can create transplantable cell sheets, including retinal pigment epithelium and corneal epithelium, using the induced pluripotent stem (iPS) cells^42,43^. However, a substantial issue in cell sheet technology is that it is still difficult to construct a three-dimensional structure of complex tissue composed of multiple cell types^33^. Periodontal tissue, which is composed of cementum, PDL and alveolar bone, is a characteristic complex tissue with a bone-ligament interface. This anatomical architecture consists a functional connection between a hard tissue and ligament structure, which enables physiological tooth function, such as the ability to alleviate excessive occlusal force, to undergo orthodontic movement via bone remodelling and perceive noxious stimulations^2,3^. In our study transplantation of PDL cell sheets regenerated with an irregularly arranged fibrous tissue formation. PDL cells consists of a group of heterogeneous cell population and consist of several cell subsets and it is known that PDL cells produce osteoblast‐related extracellular matrix proteins^44^. However, previously it was shown that there was no mineralized tissue formation in cells cultured without an osteogenic induction medium^45^. Likewise, in this study we have not used a differentiation medium for periodontal ligament cell culture or cell sheet. Our prime focus was to analyze and demonstrate the possibility of simultaneous regeneration of PDL area from PDL cells and bone area from osteoblast-like cells from a complex cell sheet fabricated using multistage layering in a temperature responsive culture dish.

A proper periodontal tissue reconstruction around the dental implant has been demonstrated with a vertical alveolar bone regeneration using the dental follicle tissues in which the layered cell differentiation has occurred^46^. For the functional regeneration of periodontal tissue, several studies indicated the importance of a biological cross-talk between bone cells and PDL cells through the fabrication of a composite cell sheet by mixing both cells^47,48^. In addition, an attractive transplantation method with the combination of multiple types of uniform cell sheets has been attempted to reproduce the periodontal tissue structure; however, the complete complex structure of natural periodontal tissue is not established^49^. In this study, we fabricated a complex cell sheet composed of bone-ligament structure by layering PDL cells and osteoblast-like cells on a temperature responsive culture dish (Figs. 2 and 3 and Supplementary data, Fig. S1). Furthermore, our complex cell sheet developed a periodontal tissue structure equivalent to natural periodontal tissue in the ectopic/orthotopic transplantation (Figs. 4–8 and Supplementary data, Figs. S4 and S5). The complex cell sheet method could be used to construct a functional bone-ligament structure as a novel technology for three-dimensional tissue regeneration.

Recent progress in regenerative technologies, which can innovate the conventional medical treatments, have been developed by incorporating stem cell biology and biomaterial sciences^50^. In the dental field, the leading technologies, such as stem cell transplantation using tissue-derived cells, cytokine therapy and scaffold engineering, have been developed, and they can be applied clinically as an effective regenerative therapy for diseases and injuries in the maxillofacial region including teeth, periodontal tissue and jaw bones^51,52^. Cell sheet technology can develop a three-dimensional regenerative tissue without artificial materials, and this scaffold-free tissue engineering has been applied clinically to various tissue regenerations^53,54^. However, a single cell sheet cannot sufficiently regenerate a large-scale tissue injury and the current technology using a uniform cell sheet has a limitation in achieving the defect size to be regenerated^33^. Many researchers and clinicians have tried to develop combination methods with uniform cell sheets and various absorbable/non-absorbable artificial scaffolds for large-scale periodontal tissue injury. The use of well-designed scaffolds, including a biphasic scaffold, fiber guiding scaffold and multiphase region-specific micro-scaffolds, were considered for three-dimensional periodontal tissue regeneration^34,55,56^. Scaffold-free tissue engineering for a large-scale defect enables complete three-dimensional tissue regeneration by applying bioengineered tissue designed to simulate exact anatomical structure. In this study, we demonstrated that our complex cell sheet laminated with PDL cells and osteoblast-like cells could achieve complete periodontal tissue regeneration including the surrounding alveolar bone in a large-scale periodontal tissue defect (Figs. 6–8). The regenerated periodontal tissue could reorganize the natural bone-ligament structure that had a functional connection with tooth root and alveolar bone (Figs. 7 and 8). This study illustrates that our complex cell sheet layered of the bone-ligament structure is a biological engineering technology that could regenerate a large-scale periodontal tissue injury without artificial materials.

The acquisition and efficient amplification of stem/progenitor cells for tissue regeneration is an essential issue for clinical application in the tissue engineering technology. In the dental field, several tooth tissue-derived stem cells have been identified, and PDLSCs and DFSCs are considered useful stem cell sources of periodontal tissue regeneration^5,46^. Furthermore, other stem cell sources including iPS cells, which are derived from the non-odontogenic cells, have been attempted for dental tissue regeneration^57,58^. For example, several studies carried out transfer of genes, such as bone morphogenic protein-2 and tumor necrosis factor-6, into iPS cells to induce differentiation of cells that could regenerate target tissue^59–62^. In addition, an effective method for functional periodontal tissue regeneration has been developed in combination with iPS cells and cytokines including EMD and growth/differentiation factor-5^63–65^. However, in current dental treatments, PDL cells and osteoblasts are the available cell sources that can be collected from living tissue and transplanted into patients. Ideally, immunological problems with transplanted tissues would be prevented by autologous transplantation of patient’s own cells, and utilization of stem cells derived from patients is currently the first choice in regenerative therapies based on tissue engineering technology^66–68^. Even though we adopted the osteoblast-like cells (MC3T3-E1 cells) to represent the bone cells in this study, in the case of clinical application using our technology, we must consider the use of osteoblasts that can be collected from the patient’s bone tissue or induce from the bone marrow stromal cells and/or PDL cells. In addition, transplantation with an excessive number of cells in a large-scale periodontal tissue injury might cause necrosis of the graft by reduced blood supply and inflammation^19^. Although in our technology using a complex cell sheet layered with multilineage cells it is important to transplant a large number of cells as a lump into the defect site, it is necessary to consider the above-mentioned problems such as necrosis and inflammation of the transplanted tissue. A previous study prevented the necrosis of transplants using a unique cell-sheet that mixed PDL cells and human umbilical vein endothelial cells (HUVECs) for periodontal tissue regeneration along with the vascular supply^34^. In addition, attractive technologies have been developed to reduce or suppress the necrosis and inflammation at the transplantation site^69,70^. The integration of these techniques into the future studies may facilitate improved bioengineered tissue engineering for the regeneration of extensive periodontal tissue defects.

In summary, our study demonstrated the fabrication of a complex cell sheet composed of PDL cells and osteoblast-like cells that anatomically reproduced the bone-ligament structure equivalent to natural periodontal tissue. Furthermore, we demonstrated three-dimensional regeneration of periodontal tissue using a complex cell sheet in a large-scale tissue injury. This study represents the feasibility of three-dimensional tissue engineering/regeneration using an anatomically designed bioengineered complex tissue.

## Materials and Methods

### Animals

All animals were purchased from a licensed company (CLEA Japan, Osaka, Japan). We used 4–5-week-old male Sprague-Dawley (SD) rats for the PDL cell collection. C57BL male mice were used for collecting mandibular first molars that were later used as a carrier for ectopic transplantation. CB17/Icr scid/scid female mice were used for ectopic transplantation and orthotopic transplantation. All of the surgical procedures were carried out under general anesthesia using 75 mg/kg medetomidine (Nippon Zenyaku Kogyo Co., Ltd, Fukushima, Japan), 4 mg/kg midazolam (Sandoz K.K., Yamagata, Japan) and 5 mg/kg butorphanol (Meiji Seika Pharma Co., Ltd., Tokyo, Japan).

### Animal ethics statement

All animal experiments were approved by the Animal Care Committee of Tokushima University (Approval No. T29-50) (Tokushima, Japan). All the animal experimental protocols used in this study was conducted in accordance with the guidelines established by the institutional ethics committee of laboratory animals.

### Cell isolation and culture

All molars of the four quadrants were carefully extracted from the 4–5-week-old male SD rats under a dissection microscope (ZEISS Stemi 508, stereo microscope). The extracted teeth were immediately immersed in Dulbecco’s modified Eagle’s medium (DMEM (High glucose), Nacalai Tesque, Inc. Kyoto, Japan), supplemented with 10% fetal bovine serum (FBS, Biowest, Ireland) and 1% antibiotic solution (penicillin-streptomycin mixed solution, Nacalai Tesque, Inc. Kyoto, Japan). Following extraction, the teeth were washed thrice with phosphate buffered saline (PBS, Takara bio Inc. Shiga, Japan) and then dispersed in 3 mg/mL Collagenase type I solution (Gibco, Grand Island, NY 14072, USA) for 30 minutes at 37 °C and the tube was slightly tapped every 5 minutes. An equal amount of culture medium was added and the supernatant collected in another tube. The cells were washed and collected 3 times using the culture medium. Centrifugation was performed at 4 degrees and 1800 rpm for 3 minutes. Finally, the culture medium containing suspended PDL cells was cultured in a 35 mm dish. PDL cells between passage 3 and 5 were used for cell sheet fabrication.

### Identification of PDL and MC3T3-E1 cells

The cell morphology was examined under a light microscope (Leica DMi 1, Wetzlar, Germany). Semi-quantitative PCR was performed to identify the genes related to each cell type. Total RNA was isolated from the cell cultures using a GenElute™ Mammalian Total RNA Miniprep Kit (Sigma Aldrich, Inc, USA). cDNA was then synthesized from 1 µg of the total RNA using a ReverTra Ace® qPCR RT Kit (Toyobo, Osaka, Japan). A PCR procedure was performed with GoTaq® Green Master Mix, 2× (Promega, Madison, WI USA) using the manufacturer’s protocol. PDL cells were identified for the gene expression of Col3a1, β-actin, Col1a2, GDF-7, scleraxis, periostin, tenascin C, fibronectin 1, β1-Integrin, CAP, f-spondin, Runx-2, OCN and ALP. MC3T3-E1 cells were identified for the gene expression of Col3a1, β-actin, Col1a2, GDF-7, periostin, tenascin C, fibronectin 1, β1-Integrin, CAP, f-spondin, Runx-2, OCN and ALP. All of the primers were designed using Bioinformatics, Primer3Plus software. The primer sequences are listed in Table 1. The PCR products were then checked by electrophoresis in 2% agarose gel with ethidium bromide.

**Table 1:** Primers used for semi-quantitative PCR for the identification of cells. (Primer No. 1 to 14 were designed for rat PDL cells and primer No. 15 to 27 were designed for MC3T3-E1 cells).

No	Gene	Primer Sequence	Product size(BP)
1.	Col3a1	Forward 5′ AAGAGCGGAGAATACTGGG3′ Reverse 5′ CAATGTCATAGGGTGCGATA3'	532
2.	β−actin	Forward 5′ GCCAACCGTGAAAAGATGAC3′ Reverse 5′ GCTCGAAGTCTAGGGCAACA3′	334
3.	Col1a2	Forward 5′ CTCGCTCACAGCCTTCACTC3′ Reverse 5′ GCTGAGTTGCCATTTCCTTG3'	396
4.	GDF-7	Forward 5′ GCCCCATCAGCATCCTCTAC3′ Reverse 5′ CAACGTCAGCAAACGAACAC3′	330
5.	Scleraxis	Forward 5′ GACCGCACCAACAGCGTGAA3′ Reverse 5′ GTGGACCCTCCTCCTTCTAACTTC3'	382
6.	Periostin	Forward 5′ CAAACCACTTTCACGGACCT3′ Reverse 5′ CAGTTTTTCGTGCAGGGACT3′	369
7.	Tenascin C	Forward 5′ GTGGATGGATCGTTTTCCTG3′ Reverse 5′ TGGCTGAGTCTGTGTCCTTG3'	336
8.	Fibronectin 1	Forward 5′ CCTTAAGCCTTCTGCTCTGG 3′ Reverse 5′ CGGCAAAAGAAAGCAGAACT 3′	301
9.	β1−Integrin	Forward 5′ GGAGGAATGTAACACGACTGC3′ Reverse 5′ CAGATGAACTGAAGGACCACC3′	701
10.	CAP	Forward 5′ TGGTGCTTTTTCTGGTCTCC3′ Reverse 5′ CGTCCTTCTCTACGATCACCTC3'	360
11.	f-spondin	Forward 5′ CTGTAATCCCAGCACTTTGG3′ Reverse 5′ GGTTCAAGCGATTCTTCTGC3'	343
12.	ALP	Forward 5′ CTACGCACCCTGTTCTGAGG3′ Reverse 5′ ATGATGGTTGCAGGGTCTGG3'	328
13.	Runx-2	Forward 5′ GGCCAGGTTCAACGATCTGA3′ Reverse 5′ GGTGGGGAGGATTGTGTCTG3'	351
14.	OCN	Forward 5′ AAGTCCCACACAGCAACTCG3′ Reverse 5′ GAAGCCAATGTGGTCCGCTA3'	301
15.	Col3a1	Forward 5′ TGGCAACCCTGGAATAGCTG3 Reverse 5′ GGGTTTCCATCCCTTCCAGG3′	306
16.	β−Actin	Forward 5′ATCGTGCGTGACATCAAAGA3′ Reverse 5′GTACTTGCGCTCAGGAGGAG3′	390
17.	Col1a2	Forward 5′ AAAGGCGTGAAAGGACACAG3′ Reverse 5′ GACCTGGAAGACCCACTTCA3'	340
18.	GDF-7	Forward 5′ GAACCAGACAGGGACAGTGC3′ Reverse 5′ CCCGGAACAGACTCTCTTTCC3'	355
19.	Periostin	Forward 5′ AACCACTTTCACCGACCTGG3′ Reverse 5′ ATGGCACCATTCCTTCCCTG3'	312
20.	Tenascin C	Forward 5′ CCACCCACTACTCAGCAAGG3′ Reverse 5′ TCCAAACCCAGCAGCATAGG3'	302
21.	Fibronectin 1	Forward 5′ GAGAACCAGGAGAGCACACC3′ Reverse 5′ AGAGGATTGCTTTCCCTGCC3'	314
22.	β1−Integrin	Forward 5′ AGTGCCATGAGGGAAATGGG3′ Reverse 5′ CAAACACGACACCTGCACAC3'	310
23.	CAP	Forward 5′ CGGGTGGTTGGTTCTTGCTA3′ Reverse 5′ TTGTACTTGTTGGGAAGCCGT3'	352
24.	f-spondin	Forward 5′ AATGTGAGAGCAGCACCCTC3′ Reverse 5′ GTGATGGACCCACCTTCTGG3'	308
25.	ALP	Forward 5′ TTCTTCTTGCTGGTGGAAGG3′ Reverse 5′ CTGGGCCTGGTAGTTGTTGT3'	353
26.	Runx-2	Forward 5′ AATGATGGTGTTGACGCTGA3′ Reverse 5′ TTGACGGTTTGTTCCTTGGT3′	323
27.	OCN	Forward 5′ CCCAGACCTAGCAGACACCA3′ Reverse 5′ CTTGCAGGGCAGAGAGAGAG3'	400

### Fabrication, histology and immunohistochemistry of cell sheets

Cell sheets were prepared using a temperature responsive culture dish (24 well culture dish, CellSeed, UpCell^®^, Tokyo, Japan). Three types of cell sheets were used for the study (Supplementary data, Fig. S1): two single cell sheets containing either PDL cells (PDL cell sheet) or MC3T3-E1 cells (MC3T3-E1 cell sheet) and a complex cell sheet containing both cells. The MC3T3-E1 cell sheet was fabricated at a seeding density of 1 × 10^6^ cells per well and cultured in α-minimum essential medium (α-MEM) (Nacalai Tesque, Inc. Kyoto, Japan) with 10% FBS and 1% antibiotics. After 2 days, the medium was changed to osteogenic induction medium containing α-MEM supplemented with 10% FBS, 10 nM dexamethasone (Sigma Aldrich, Inc, USA), 50 µg/ml ascorbic acid (Sigma Aldrich, Inc, USA) and 5 Mm β-glycerophosphate (Sigma Aldrich, Inc, USA) for 2 more days. After 3–5 days, the cell sheets were collected. The PDL cell sheet was made by seeding 1 × 10^6^ PDL cells per well and culturing in DMEM supplemented with 10% FBS and 1% antibiotic solution for up to 48 hours. The complex cell sheet was fabricated by layering PDL cells on top of the MC3T3-E1 cell culture. Firstly, the MC3T3-E1 cell sheet was cultured at a seeding density of 5 × 10^5^ cells per well and cultured in α-MEM supplemented with 10% FBS and 1% antibiotics. After 2 days, the medium was changed to osteogenic induction medium containing α-MEM supplemented with 10% FBS, 10 nM dexamethasone, 50 µg/ml ascorbic acid and 5 mM β-glycerophosphate for 2 more days. The culture well was then carefully washed once with warm PBS to remove the osteogenic induction medium. 1 × 10^6^ PDL cells were plated on top of the MC3T3-E1 cell sheet in the same temperature responsive culture wells. Complex cell sheets were grown in DMEM supplemented with 10% FBS and 1% antibiotic solution for up to 48 hours. All the cell sheets were harvested by reducing the temperature.

After overnight fixation in a 4% paraformaldehyde phosphate buffer solution (Nacalai Tesque, Inc. Kyoto, Japan), cell sheets were embedded in paraffin and cut into 5 µm sections. The samples were dewaxed, rehydrated and stained with hematoxylin-eosin (H&E) for morphological observation. For immunohistochemical analysis, cell sheet sections were dewaxed and dehydrated using graded concentrations of ethanol. The samples were then immersed in a combined solution of 0.3% H_2_O_2_ in methanol for 30 minutes. The samples were washed with Tris-buffered saline (TBS, pH 7.6). To avoid any non-specific binding, samples were blocked with Protein Block Serum-Free (Dako) for 30 minutes. The sections were incubated overnight with the primary anti-mouse-OCN antibody (1:200) (Abcam, Tokyo, Japan). Further processing of immunohistochemistry was performed using the Vectastain ABC kit (rabbit IgG; Vector Laboratories). The antigen reaction was visualized using a 3,3-diaminobenzidine (DAB; Sigma-Aldrich, Tokyo, Japan) chromogenic substrate, counterstained with Mayer’s hematoxylin and observed under a light microscope.

### Fluorescent *in situ* hybridization (FISH) for complex cell sheet

FISH technique was performed according to the manufacturers protocol. Briefly, the paraffin sections were dewaxed, rehydrated, then incubated in 0.5% pepsin/0.1 N HCL solution at 37 °C for 10 minutes. The slides were then washed in PBS once, followed by alcohol dehydration. The probe (SPR-20, species specific FISH probe, Rat (Cy3) (CSL, Chromosome science, Japan) was applied to the specimen and incubated at 80 °C for 10 minutes, followed by overnight hybridization at 37 °C. The following day, slide was immersed in 2X saline sodium citrate buffer (2X SSC), then incubated in 50% formamide/ 2X SSC at 37 °C for 20 minutes. Finally, the slide was mounted using Fluoro-KEEPER Antifade Reagent, Non-Hardening Type with DAPI (Nacalai Tesque, Kyoto, Japan) and fluorescence detection were performed using a Nikon A1 laser scanning confocal microscope (Nikon Instruments Inc. Melville, U.S.A).

### Alizarin red staining of complex cell sheets

Following overnight fixation in a 4% paraformaldehyde phosphate buffer solution (Nacalai Tesque, Inc. Kyoto, Japan), cell sheets were embedded in paraffin and cut into 5 µm sections. The samples were dewaxed, rehydrated and stained with stained with 1% Alizarin red solution (Muto Pure Chemicals Co., Japan) for 10 min and analyzed under light microscope.

### Ectopic transplantation

The PDLs on the extracted mice mandibular first molar teeth were removed using enzymatic digestion with collagenase type I solution, and were then autoclaved to remove the remnants of any cells in the tooth (Supplementary data, Fig. S2). These teeth were used as a carrier for ectopic transplantation of the cell sheets. CB17/Icr-scid/scid female mice were used as a host for subrenal capsule transplantation. Cell sheets were sectioned into 4 pieces and each section was wrapped around the mice mandibular first molar roots. This tooth cell sheet complex was inserted into collagen gel (Cellmatrix®, Nitta Gelatin Inc.) and incubated at 37 °C for 20 minutes to coagulate the collagen gel. Subrenal capsule transplantation of the cell sheet unit was performed as described previously^71^. All samples were transplanted into the subrenal capsule of immunocompromised mice with each mice receiving 4 transplants. PDL cells side of  the complex cell sheet were facing the root surface. The procedure was carried out under a dental microsurgery microscope (Leica M 320 F 12). The transplants were collected after 4 weeks (Supplementary data, Fig. S2).

### Orthotopic transplantation

A periodontal tissue injury defect in CB17/Icr-scid/scid female mice were used. A small incision was made from the mesial side of the maxillary first molar and the palatal mucosa was gently elevated up to the maxillary second molar. The periodontal tissue injury was created on the palatal bone overlying the maxillary first molars using a small round carbide burr (ZIPPER carbide burr, HP 1/4) at a slow speed. The procedure was carried out under a dental microsurgery microscope (Leica M 320 F 12). The injury was carefully created by removing the bone and PDL adjacent to the maxillary first molar tooth without trimming the root. The defect was created from the cervical portion of the crown and the palatal roots were exposed (Fig. 6B (shown by red arrows) & Fig. S3A,B). The defect area was irrigated with abundant PBS and wiped using sterile cotton rolls. Cell sheets were trimmed to the appropriate shape and adapted to the exposed root surface. As we explained in the ectopic transplantation, the PDL cell side of complex cell sheet was facing towards the root surface and the MC3T3-E1 cell side towards the bone tissue side. The mucosa was sutured back in position with 8-0 nylon (Bear medic, Chiba, Japan).

### Microcomputed radiographic imaging and bone regeneration volume analysis using micro computed tomography (micro-CT)

After 8 weeks of orthotopic transplantation, all animals were sacrificed by cervical dislocation. Surgical sites were dissected, washed once in PBS and then fixed in 4% paraformaldehyde phosphate buffer solution for 2 days and analyzed using micro-CT with SkyScan 1176 (SkyScan, Kontich, Belgium). Scanning was performed at 65 kV and 380 µA at a reconstruction angular range of 360° under a voxel size of 9 µM. The scanned images were reconstructed using NRecon software (Bruker micro CT, Kontich, Belgium). Three-dimensional modeling analysis of reconstructed images were carried out using CTVox software (Bruker micro CT, Kontich, Belgium). Region of interest (ROI) was selected from the palatal bone adjacent to the first molar tooth on right and left side (Fig. S3C,D, is indicated in blue color) and regenerated bone volume at the injury site was analyzed and quantified using CT analyzer software (CTAn, Bruker micro CT, Kontich, Belgium).

### Histological and immunohistochemical analysis of transplants

Ectopic and orthotopic transplant samples were decalcified in 8% ethylene diamine tetracetic acid (EDTA) solution for 3–4 weeks. After decalcification, samples were embedded in paraffin for 5 µm histologic sectioning. For histologic analysis, ectopic and orthotopic transplant sections were stained with H&E or azan staining. For immunohistochemical analysis, ectopic and orthotopic transplant tissue sections were dewaxed and dehydrated using graded concentrations of ethanol. The samples were then immersed in a combined solution of 0.3% H_2_O_2_ in methanol for 30 minutes. The samples were washed with Tris-buffered saline (TBS, pH 7.6). To avoid any non-specific binding, samples were blocked with Protein Block Serum-Free (Dako) for 30 minutes. The sections were incubated overnight with the primary anti-periostin antibody (1:500) (Abcam, Tokyo, Japan) or anti-mouse-OCN antibody (1:200) (Abcam, Tokyo, Japan). Further processing of immunohistochemistry was performed using the Vectastain ABC kit (rabbit IgG; Vector Laboratories). The antigen reaction was visualized using a 3,3-diaminobenzidine (DAB; Sigma-Aldrich, Tokyo, Japan) chromogenic substrate, counterstained with Mayer’s hematoxylin and observed under a light microscope (OlympusBX50 upright microscope, DP70 microscope digital camera, Tokyo, Japan).

### Statistical analysis

The experimental values were expressed as means ± SD. Statistical significance was evaluated by one-way ANOVA followed by the Tukey HSD test. The significance level was set at *P < *0.05.

## Supplementary information

Dataset 1.
