# Supplementary material for: Three-dimensional periodontal tissue regeneration using a bone-ligament complex cell sheet
Source: Sci Rep. 2020 Feb 3;10:1656. doi: 10.1038/s41598-020-58222-0 (PMC6997427; doi:10.1038/s41598-020-58222-0)
Supplement: Supplementary file 1 — Dataset 1. [file 41598_2020_58222_MOESM1_ESM.pdf]

Supplementary Information

Three-dimensional periodontal tissue regeneration using a bone-ligament complex cell sheet

Resmi Raju[1], Masamitsu Oshima[1], Miho Inoue[1], Tsuyoshi Morita[2], Yan Huijiao[1], Arief Waskitho[1], Otto Baba[2], Masahisa Inoue[3], Yoshizo Matsuka[1,*]

[1]*Department of Stomatognathic Function and Occlusal Reconstruction, Graduate School of Biomedical Sciences, Tokushima University, Tokushima, 770-8503, JAPAN*
[2]*Department of Oral and Maxillofacial Anatomy, Graduate School of Biomedical Sciences, Tokushima University, Tokushima, 770-8503, JAPAN*
[3]*Laboratories for Structure and Function Research, Faculty of Pharmaceutical Sciences, Tokushima Bunri University, Tokushima, 770-8055, JAPAN*

* To whom correspondence may be addressed: Yoshizo Matsuka, DDS, PhD
Professor and Chair, Department of Stomatognathic Function and Occlusal Reconstruction, Graduate School of Biomedical Sciences, Tokushima University, Tokushima, 770-8503, JAPAN
Phone: +81-88-633-7350, Fax: +81-88-633-7391,
E-mail: matsuka@tokushima-u.ac.jp

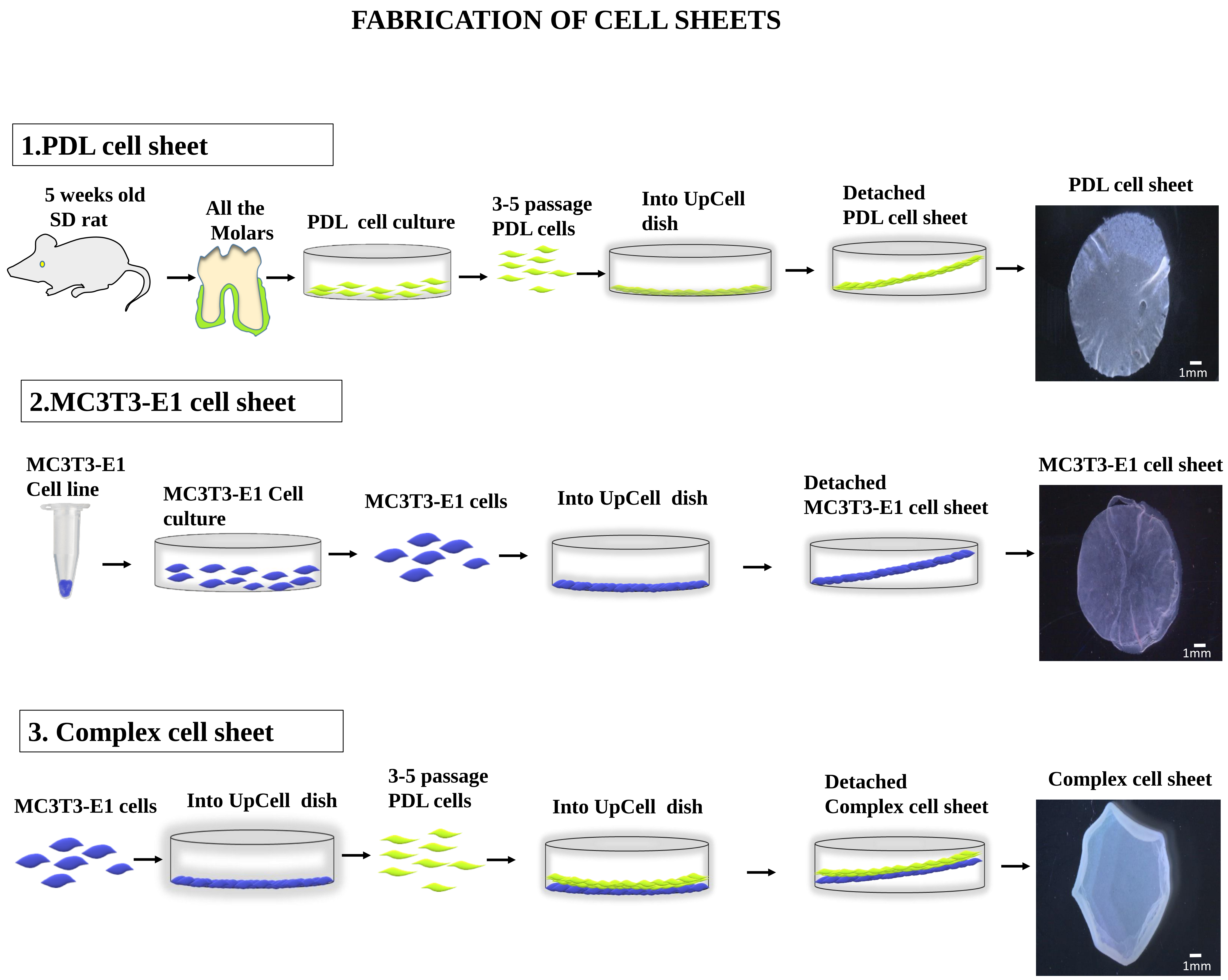

Fig. S1. Schematic representation of protocol used to fabricate 3 types of cell sheets

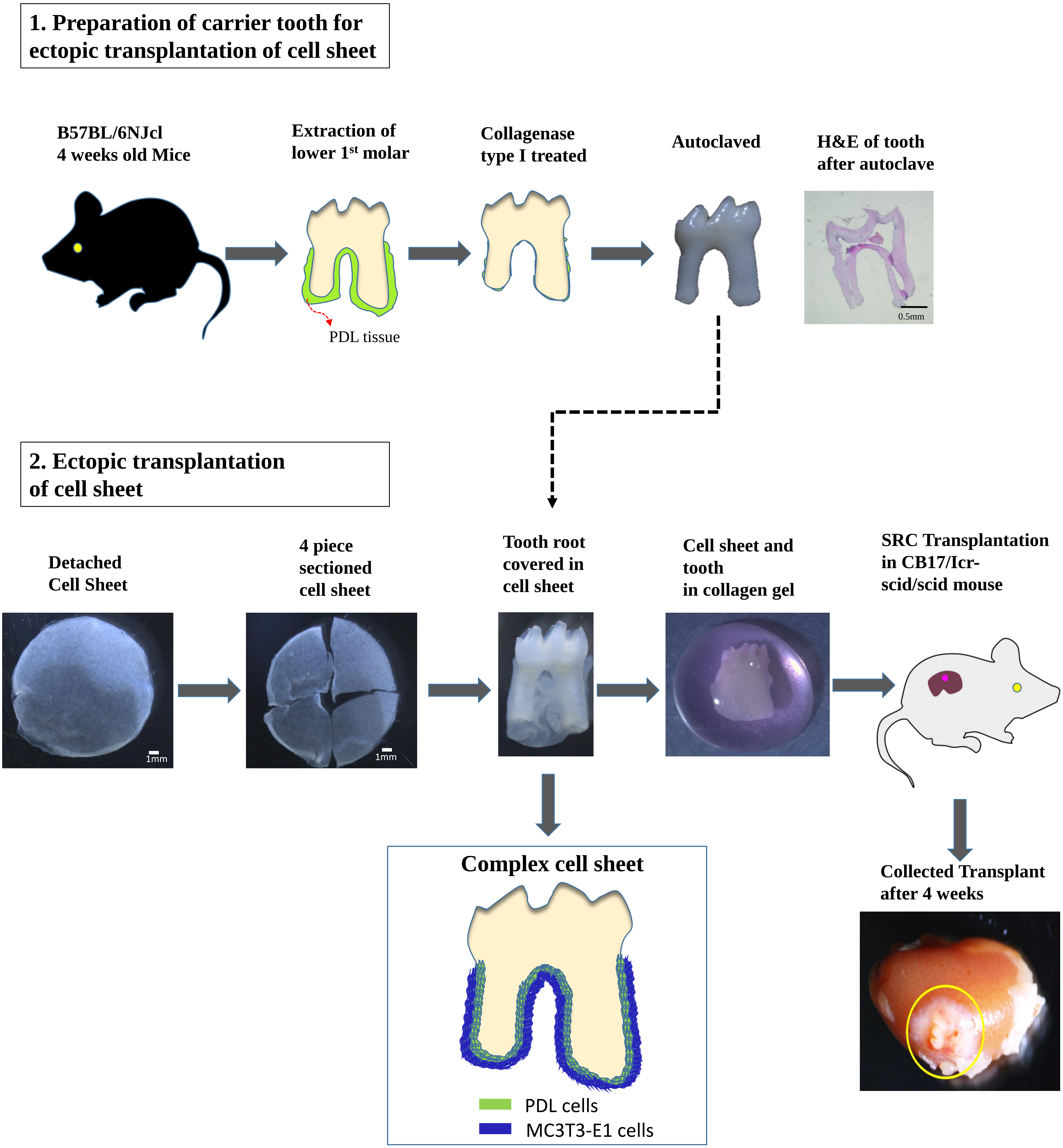

Fig. S2. Schematic representation of the protocol used in ectopic transplantation of cell sheets

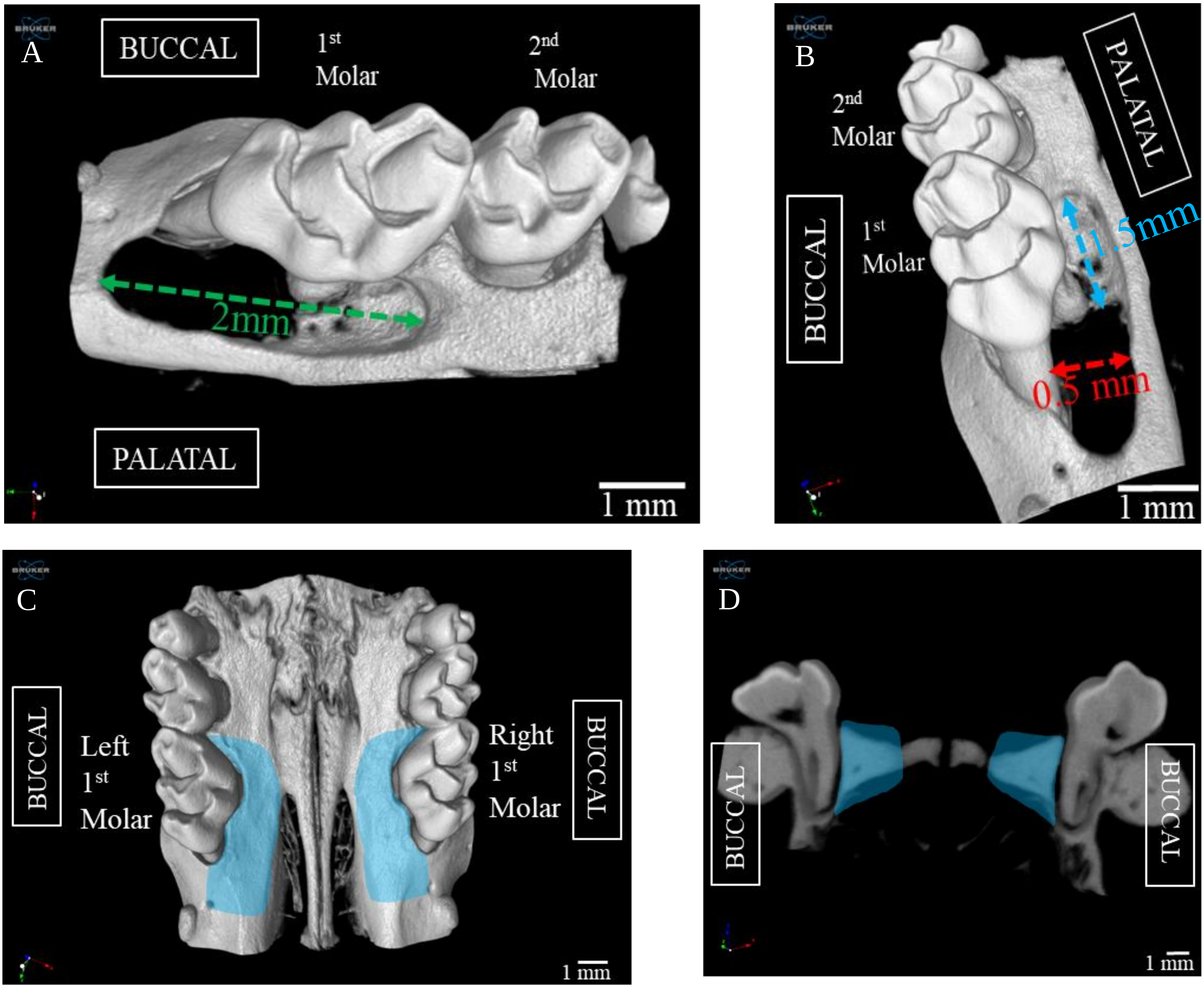

Fig. S3. Three dimensional micro-CT images showing the periodontal tissue injury size and region of interest (ROI) for bone volumetric analysis. (A) Length (anterior-posterior direction) of the periodontal tissue injury defect is 2 mm (Indicated in green double headed dotted arrow points), (B) Width (buccal-palatal direction) of periodontal injury site is 0.5 mm (Indicated in red double headed dotted arrow points) and depth of periodontal tissue injury site is 1.5 mm (Indicated in blue double headed dotted arrow points), (C) Selected ROI in anterior-posterior direction is indicated as blue color, (D) Selected ROI in buccal-palatal direction and depth is indicated in blue color.

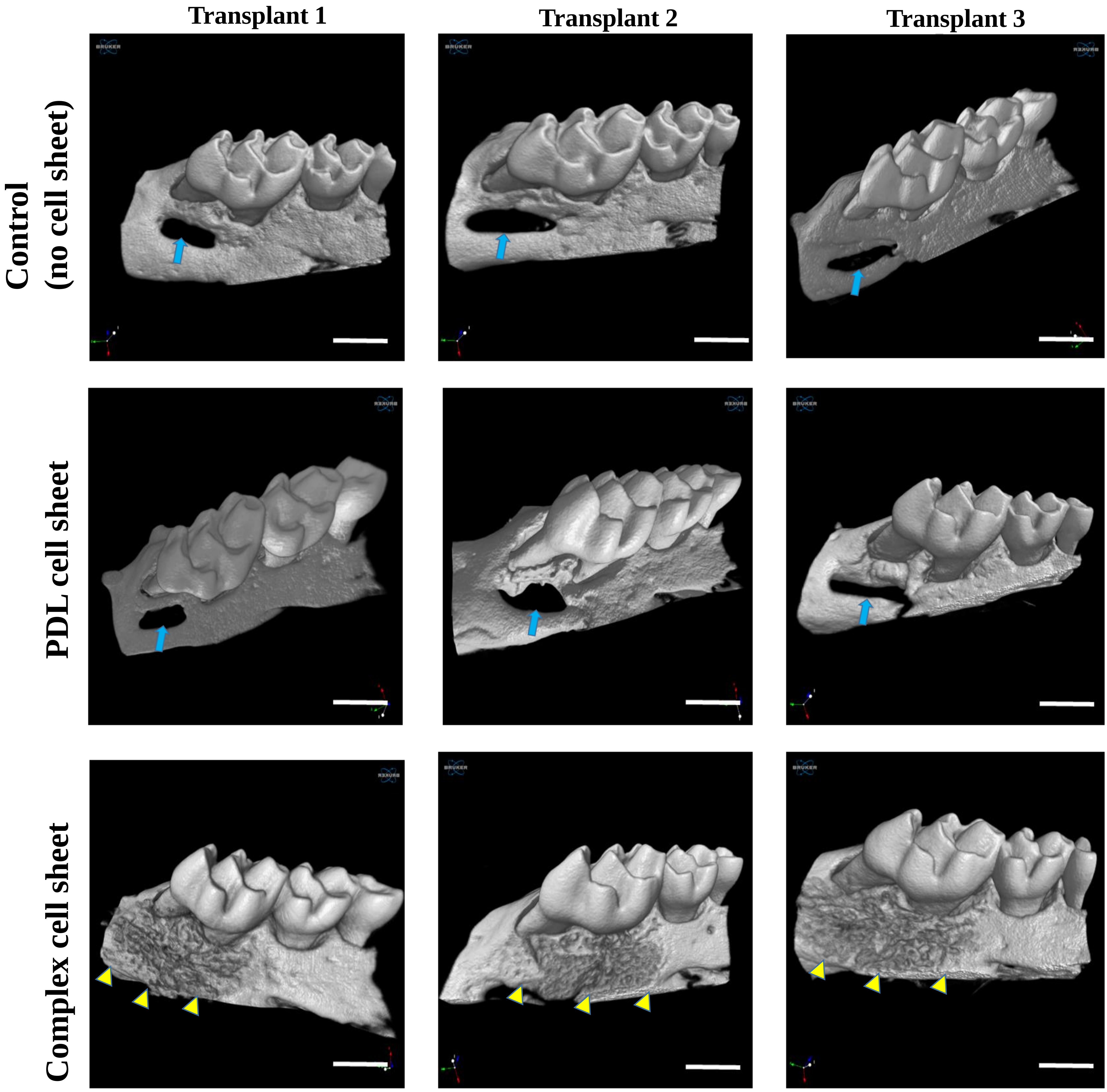

Fig. S4. Three dimensional micro-CT images showing the orthotopic transplants (3 transplants in each group). Control, PDL cell sheet and complex cell sheet transplants after 8 weeks (remaining defect after 8 weeks is indicated in blue arrow points and regenerated area is indicated in yellow arrow heads), Scale bar represents 1mm.

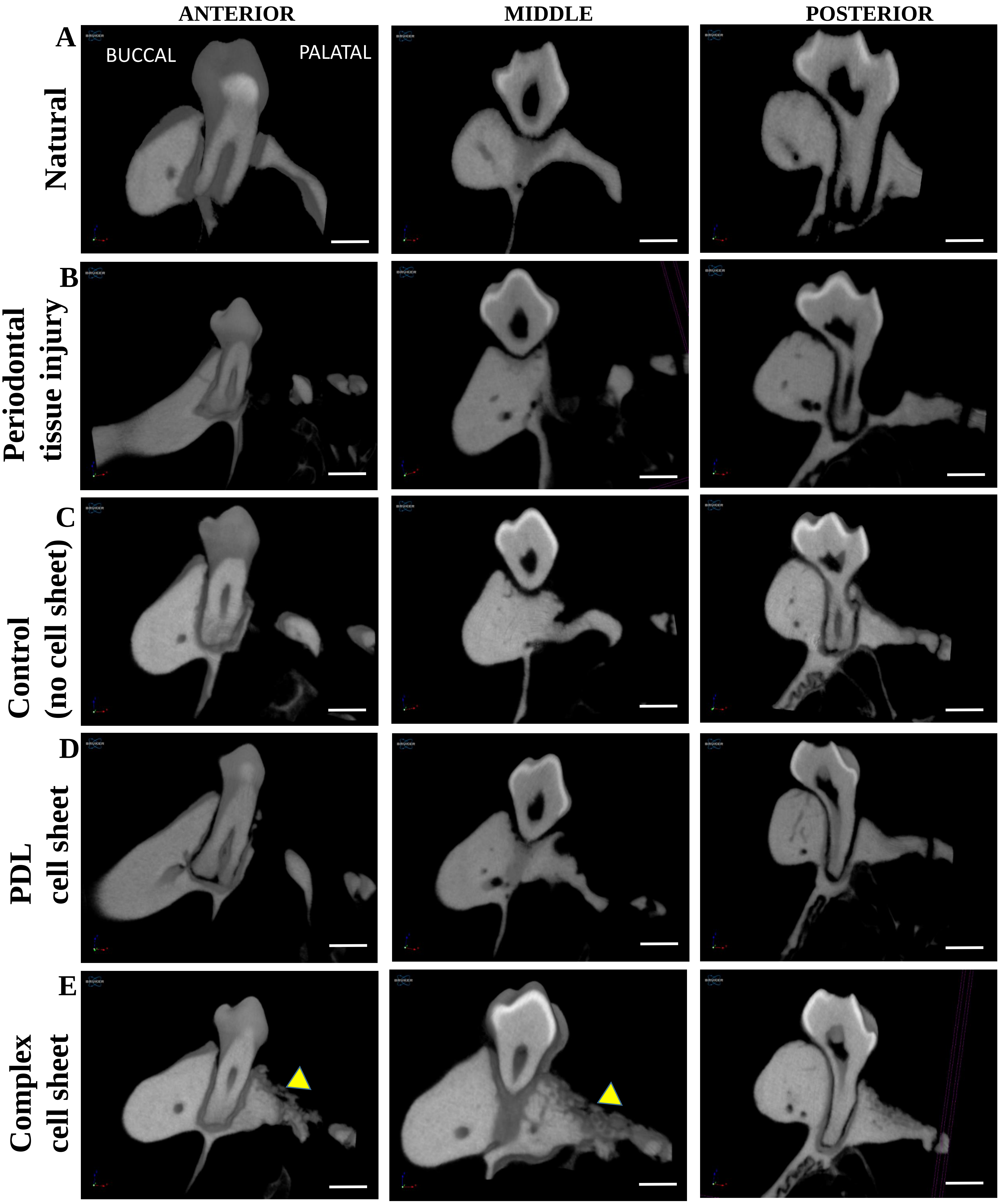

Fig. S5. Micro-CT images of orthotopic periodontal tissue injury models at anterior, middle and posterior coronal sections. (A) Natural periodontal tissue, (B) Periodontal tissue injury on day 1, (C) Control group after 8 weeks, (D) PDL cell sheet transplant after 8 weeks, (E) Complex cell sheet transplant after 8 weeks (Yellow arrowhead shows the regenerated area in complex cell sheet group). Scale bar indicates 1mm.
